# Efficacy of IPACK block combined with intra-articular steroid injection in advanced knee osteoarthritis: a retrospective cohort study

**DOI:** 10.1186/s12871-026-03896-8

**Published:** 2026-05-07

**Authors:** Halil Ibrahim Altun, Salim Taner Gözükızıl

**Affiliations:** 1 Department of Anesthesiology, Division of Pain Medicine, Kanuni Sultan Süleyman Training and Research Hospital, Atakent Mh, Turgut Ozal Bulvari No:46/1, Kucukcekmece, Istanbul, Türkiye; 2 Department of Pain Medicine, Prof. Dr. Cemil Taşcıoglu City Hospital, Darülaceze Cad. No:27 Şişli, Istanbul, Türkiye

**Keywords:** Knee osteoarthritis, Intra-articular injection, Steroid, IPACK block, Nerve block, Ultrasound guidance

## Abstract

**Background:**

Knee osteoarthritis is a major cause of chronic pain and functional limitation, particularly in advanced stages. Intra-articular steroid injections and genicular nerve interventions are commonly used for pain control; however, these methods mainly target the anterior knee and may not sufficiently relieve pain originating from the posterior capsule. The infiltration between the popliteal artery and the capsule of the knee (IPACK) block is a regional anesthesia technique that provides posterior knee analgesia without causing motor weakness. Evidence regarding its effectiveness in chronic, non-surgical knee osteoarthritis is limited. This study aimed to evaluate whether adding an ultrasound-guided IPACK block to intra-articular steroid injection improves pain and functional outcomes in patients with advanced knee osteoarthritis.

**Methods:**

This retrospective observational cohort study included 98 patients with Kellgren–Lawrence stage 3–4 knee osteoarthritis who had persistent pain despite conservative treatment. Patients received either intra-articular steroid injection alone (IASI group, *n* = 50) or intra-articular steroid injection combined with an IPACK block (IASI+IPACK group, *n* = 48). Pain severity was assessed using the Numeric Rating Scale (NRS), and functional status was evaluated with the Western Ontario and McMaster Universities Osteoarthritis Index (WOMAC). Evaluations were performed at baseline, 1 month, and 6 months after the procedure. Non-parametric tests were used for statistical analysis, and *p* < 0.05 was considered significant.

**Results:**

Both groups showed significant improvement in NRS and WOMAC scores at 1 and 6 months compared with baseline (*p* < 0.05). The IASI+IPACK group demonstrated greater pain reduction at 1 month and significantly better WOMAC Function and Total scores at both follow-up visits compared with the IASI group (*p* < 0.05). Although scores increased slightly between 1 and 6 months in both groups, outcomes remained improved compared with baseline. No major complications were observed during the follow-up period. However, minor adverse events were not systematically recorded due to the retrospective nature of the study.

**Conclusions:**

Adding an IPACK block to intra-articular steroid injection may provide additional benefit in early pain relief and functional improvement in patients with advanced knee osteoarthritis. This combined approach appears to be a safe and useful option for patients who are not surgical candidates or are awaiting arthroplasty.

**Trial registration:**

ClinicalTrials.gov, NCT07269444. Retrospectively registered on 20 November 2025.

## Introduction

Knee osteoarthritis (OA) is a multifactorial chronic joint disease affecting not only cartilage but also the meniscus, ligaments, and periarticular structures. It is one of the leading causes of chronic pain and disability worldwide [1]. Symptomatic knee OA affects approximately 10–13% of individuals over the age of 60, with increasing prevalence due to aging populations and rising obesity rates [2]. Pain, stiffness, and functional limitation significantly impair quality of life and are associated with substantial socioeconomic burden, particularly in advanced stages [3–5].

Management of knee osteoarthritis ranges from conservative treatments to intra-articular interventions and surgery; however, pain control remains challenging in non-surgical patients. While intra-articular steroid injections and genicular nerve blocks are commonly used, they may not adequately address posterior knee pain [6, 7]. Standard genicular blocks typically target the anterior and lateral aspects of the knee, often neglecting the innervation of the posterior region. This limitation highlights the need for novel methods that can provide more comprehensive and effective analgesia.

The IPACK block is a relatively novel regional anesthesia technique that provides posterior knee analgesia without causing motor blockade by targeting terminal articular branches while sparing major nerves [8–10]. While its efficacy in multimodal analgesia following knee surgery is supported by randomized controlled trials [11], this method also may be useful as an alternative treatment for patients with advanced knee osteoarthritis. The combined anti-inflammatory effect of the steroid injection and the posterior sensory blockade provided by the IPACK block may create a complementary or possibly synergistic benefit, leading to longer-lasting relief. Although the role of the IPACK block in post-arthroplasty pain management has been extensively studied, data regarding its efficacy in non-surgical chronic pain treatment are limited. The aim of this study was to evaluate whether adding an IPACK block to intra-articular steroid injection improves pain and functional outcomes in patients with advanced knee osteoarthritis.

## Methods

### Study design and patients

This retrospective observational cohort study was conducted between January 2024 and September 2025 at a tertiary hospital in Istanbul, Turkey, based on the review of electronic medical records. This study was conducted in a non-surgical setting focusing on chronic pain management rather than perioperative care. The study was designed and reported in accordance with the Strengthening the Reporting of Observational Studies in Epidemiology (STROBE) guidelines. The study was approved by the Institutional Ethics Committee (385/20.11.2025), and the study was registered in the ClinicalTrials.gov database (NCT07269444). The research was conducted in full compliance with the ethical principles outlined in the 2024 revision of the Declaration of Helsinki. While routine written informed consent for the clinical procedures was obtained from all patients, the requirement for study-specific informed consent was waived by the ethics committee due to the retrospective design of the study.

The decision to perform intra-articular steroid injection alone or in combination with an IPACK block was based on the clinical judgment of the treating physician. In general, patients presenting with more prominent posterior knee pain, inadequate response to prior conservative treatments, and greater functional limitation were more likely to receive the combined intervention.

Patients aged 40 years and older diagnosed with Kellgren-Lawrence stage 3 or 4 knee osteoarthritis, who had a Numeric Rating Scale (NRS) score greater than 4 despite prior conservative medical and physical therapy, were screened for eligibility. Only patients with Kellgren–Lawrence stage 3–4 osteoarthritis were included in order to ensure a relatively homogeneous population with advanced disease requiring interventional pain management, as patients in earlier stages are more likely to respond to conservative treatment. Exclusion criteria included a history of knee surgery, knee injections within the last six months, psychotic disorders, bleeding diathesis, active malignancy or infection, known allergies to the study medications, and inability to communicate or refusal to undergo the procedure. The diagnosis of knee osteoarthritis was confirmed by clinical examination and radiological imaging, including magnetic resonance (MRI) or plain radiography (X-RAY).

### Procedures

All injections were performed by a single physician experienced in musculoskeletal interventions, using a Toshiba TUS-A300™ (USA) ultrasound device. All procedures were carried out under aseptic conditions, and local anesthesia was applied to the subcutaneous tissue prior to needle insertion.

#### Intra-articular Steroid Injection (IASI)

The patient was positioned supine with the knee in slight flexion (approximately 15–30 degrees) and supported. A high-frequency linear ultrasound probe was placed transversely superior to the patella to visualize the suprapatellar recess. Using an in-plane technique, a 22G spinal needle was advanced from the lateral aspect toward the suprapatellar pouch, visualized as the hypoechoic space between the prefemoral fat pad and the quadriceps tendon. Once the needle tip was confirmed to be within the joint space, a mixture of 4 ml of 1% lidocaine and 40 mg of triamcinolone acetonide was injected without resistance. The spread of the injectate within the joint was verified under real-time ultrasound guidance.

#### IPACK block

**Fig. 1:**
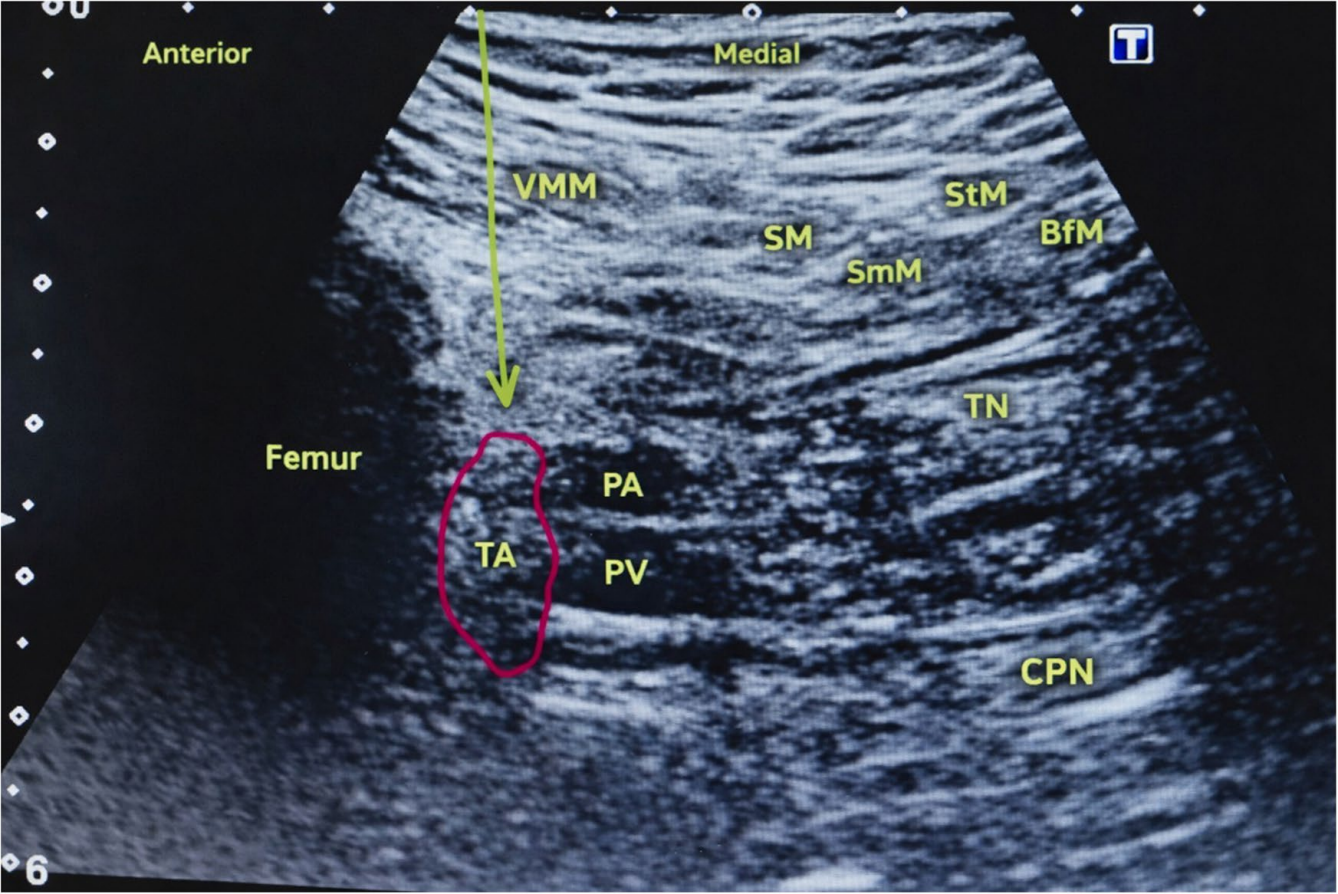
Ultrasound-guided IPACK block demonstrating the Target area (TA) and surrounding anatomical structures. PA: Popliteal artery, PV: Popliteal vein, TN: Tibial nerve, CPN: Common peroneal nerve, VMM: Vastus medialis muscle, SM: Sartorius muscle, StM: Semitendinosus muscle, SmM: Semimembranosus muscle, BfM: Biceps femoris muscle

With the patient in the supine position, the leg was placed in the “frog-leg” position (knee flexed and hip externally rotated). A low-frequency convex ultrasound probe was placed transversely over the popliteal fossa, approximately 2–3 cm proximal to the femoral condyles. The probe was adjusted proximally and distally to obtain an optimal view of the distal femoral shaft and the popliteal artery. Targeting the space between the femur and the popliteal artery, a 20G block needle was advanced from anteromedial to posterolateral using an in-plane technique. After positioning the needle tip approximately 1–2 cm beyond the lateral border of the popliteal artery, a mixture of 15 ml of 0.25% bupivacaine and 4 mg of dexamethasone was injected during needle withdrawal following negative aspiration (Fig. 1).

Following the procedure, patients were observed for two hours to monitor for potential complications. One week after the injection, all patients were routinely provided with a standardized home exercise program and were instructed by healthcare professionals on how to perform the exercises properly. However, adherence to the home exercise program was not objectively monitored due to the retrospective design of the study. Patients were advised to use 500 mg of paracetamol (up to a maximum of four tablets per day) as a rescue analgesic only if the pain became intolerable. To ensure the reliability of clinical assessments, they were instructed to refrain from taking analgesics on the days of their follow-up visits.

### Outcome measures

Baseline demographic data (age, gender, body mass index [BMI], affected side, Kellgren-Lawrence stage) and symptom duration were recorded for all included patients. All patients had previously undergone conservative management, including pharmacological treatment and physical therapy; however, detailed information regarding the duration and specific modalities of these treatments was not consistently available due to the retrospective study design. To monitor treatment efficacy, all clinical assessments were performed at baseline (pre-procedure), and at 1-month and 6-month follow-up visits post-procedure.

#### Primary outcome measure

Pain severity was assessed using the Numeric Rating Scale (NRS), an 11-point scale ranging from 0 (no pain) to 10 (worst possible pain). NRS scores during activity were recorded for evaluation. The primary outcome measure was defined as the change in NRS scores during activity between baseline and follow-up time points (1st and 6th months).

#### Secondary outcome measures

Physical functional status and limitations in daily living activities were evaluated using the Western Ontario and McMaster Universities Osteoarthritis Index (WOMAC), for which the validity and reliability of the Turkish version have been established [12]. Higher WOMAC scores indicate increased pain and functional impairment, whereas lower scores reflect clinical improvement.

Patients were advised to use 500 mg of paracetamol as a rescue analgesic only when pain was intolerable during the post-procedure period. The recorded data were based on patient self-report and could not be independently verified. Daily average paracetamol consumption (mg/day) was recorded at follow-up intervals (1 month and 6 months post-procedure). Potential complications such as infection at the injection site, hematoma, neurological deficits, or systemic reactions were also monitored throughout the follow-up period.

### Statistical analysis

Statistical analyses were performed using SPSS software version 27.0 (IBM Corp., Armonk, NY, USA). Descriptive statistics were presented as median (interquartile range) for continuous variables and as frequencies and percentages for categorical variables. The normality of data distribution was assessed using the Kolmogorov-Smirnov test. Since the data did not follow a normal distribution, non-parametric tests were utilized for analyses.

Comparisons of demographic data and treatment outcomes between the two independent groups were conducted using the Mann-Whitney U test. The homogeneity of categorical variables between groups was analyzed using the Chi-square test. Within-group comparisons of repeated measures (pre-procedure vs. post-procedure) over time were performed using the Wilcoxon Signed-Rank Test. A p-value of < 0.05 was considered statistically significant for all tests. Accordingly, continuous variables were presented as median and interquartile range.

## Results

**Fig. 2:**
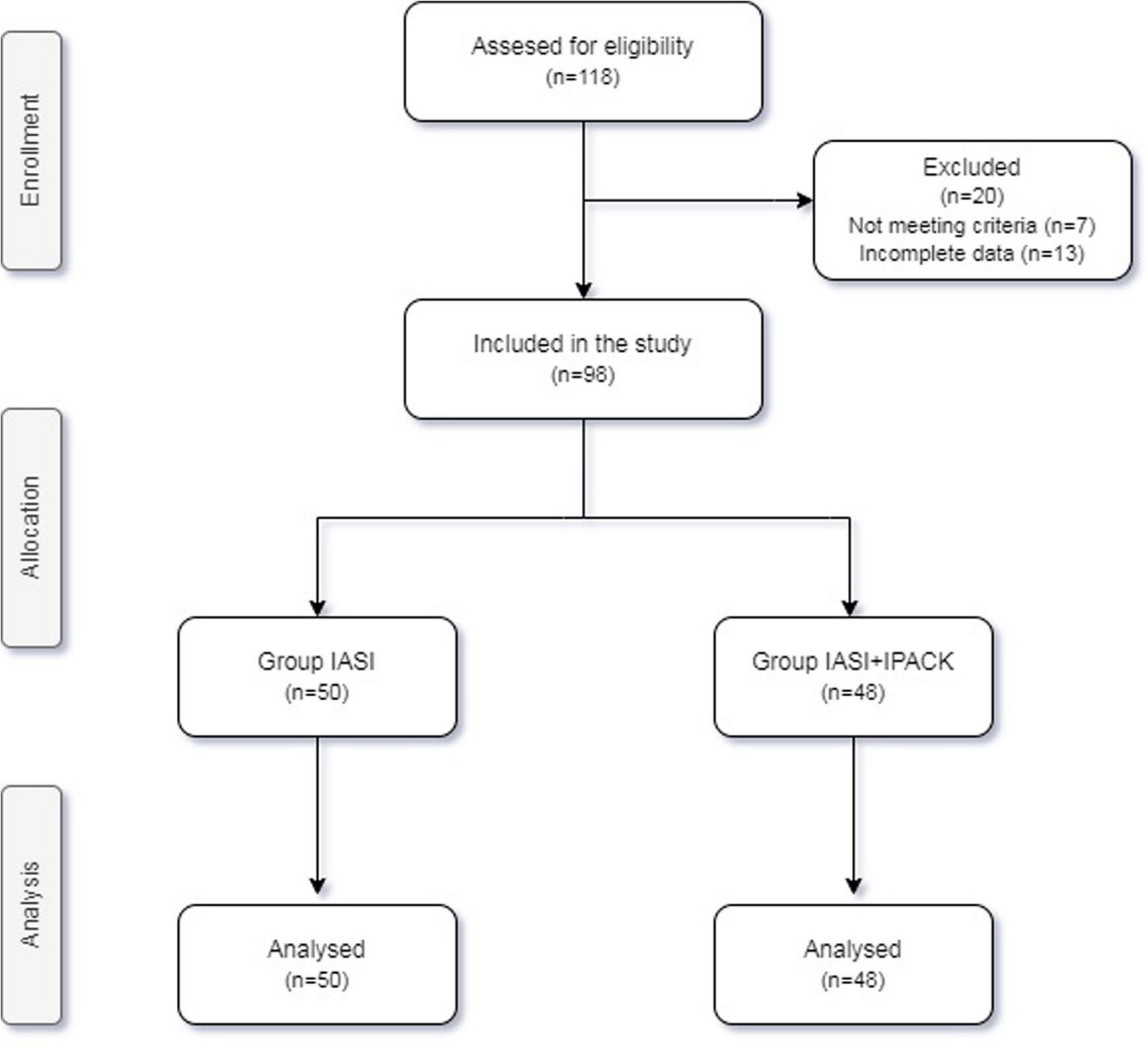
Flowchart of study. IASI: Intra Articular Steroid Injection IPACK: Infiltration between the Popliteal Artery and Capsule of the Knee

Overall, 118 patients were assessed for eligibility. After excluding 7 patients who met the exclusion criteria and 13 patients with incomplete follow-up data, a total of 98 patients were included in the final analysis. Patients were divided into two groups: the Intra-articular Steroid Injection only group (Group IASI; *n* = 50) and the group receiving Intra-articular Steroid Injection combined with an IPACK block (Group IASI+IPACK; *n* = 48) (Fig. 2).

Of the included patients, 65 (66.3%) were female, 56 (57.1%) had Stage 4 osteoarthritis, and 56 (57.1%) presented with symptoms in the right knee (Table 1). Baseline demographic and clinical characteristics were similar between the groups. No statistically significant differences were observed in age, gender, BMI, symptom duration or baseline pain/function scores (*p* > 0.05).

**Table 1:** Demographic and clinical characteristics

Injection	*n*	%
IASI IASI+IPACK Total	50 48 98	51,0 49,0 100,0
Sex		
Woman Man Total	65 33 98	66,3 33,7 100,0
Side		
Right Left Total	56 42 98	57,1 42,9 100,0
Kellgren-Lawrence		
3 4 Total	42 56 98	42,9 57,1 100,0

*IASI* Intra Articular Steroid Injection, *IPACK* Infiltration between Popliteal Artery and Capsule of the Knee

### Within-group changes

**Fig. 3:**
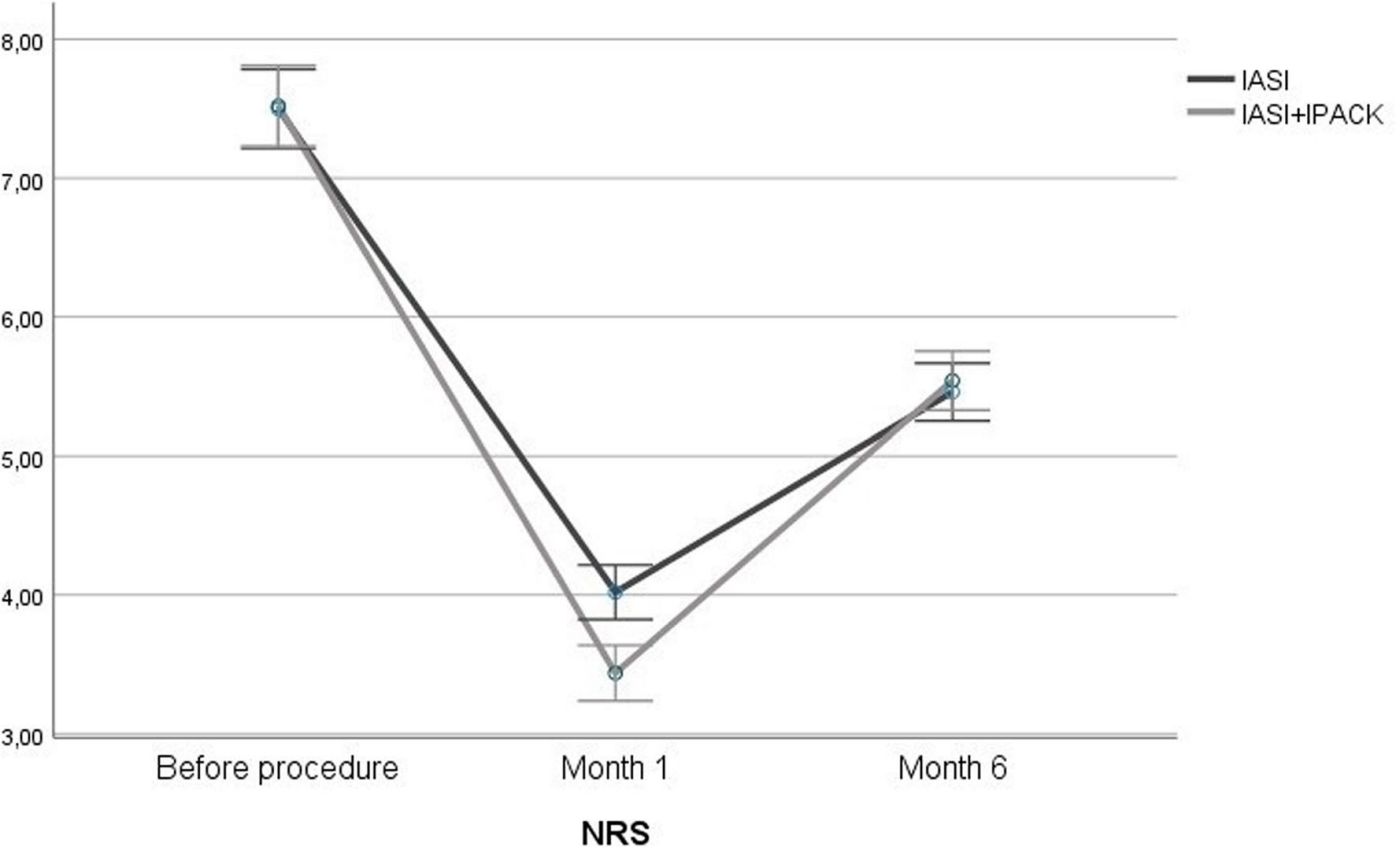
Changes in Numeric Rating Scale (NRS) scores over time in both groups

**Fig. 4:**
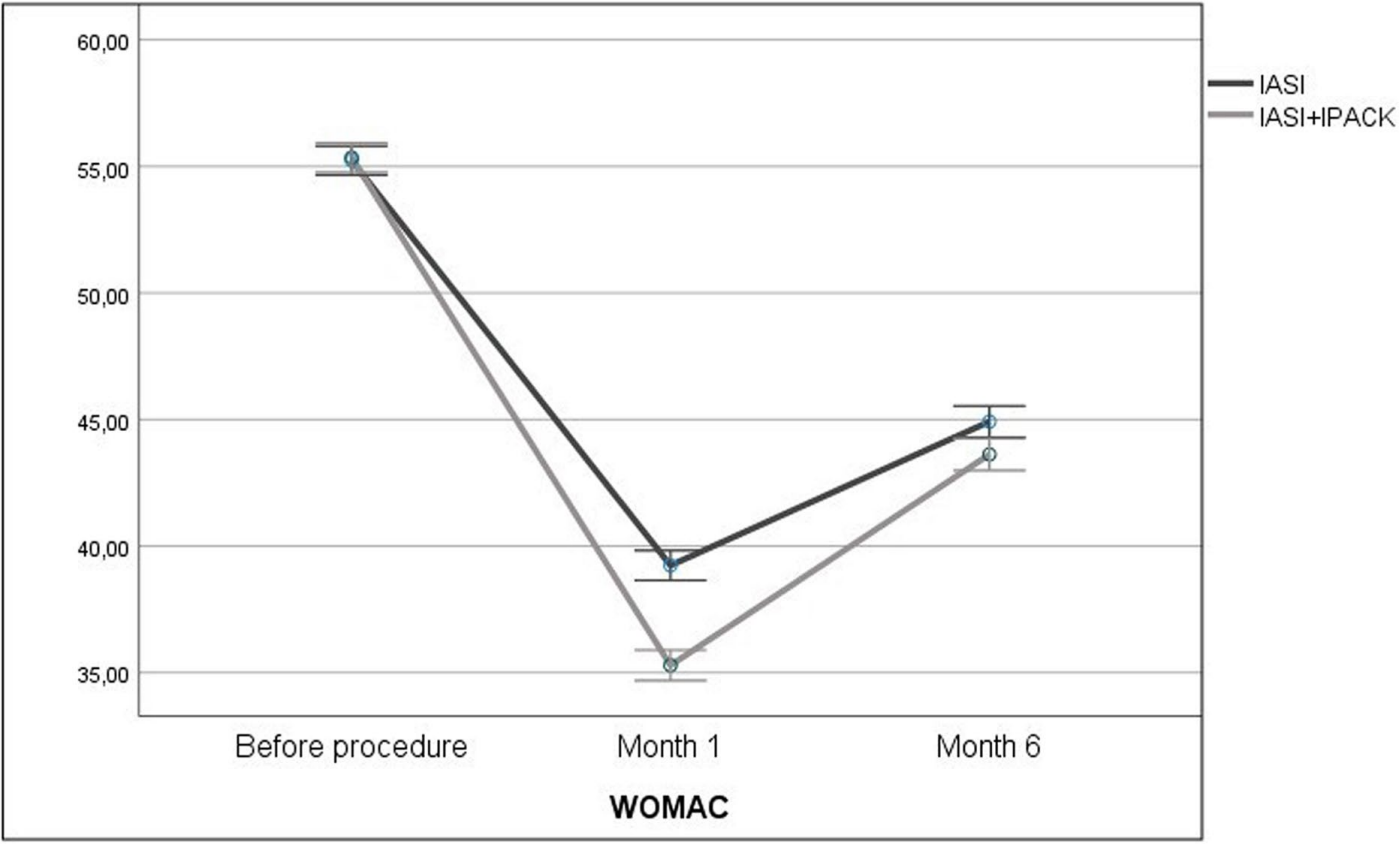
Changes in WOMAC total scores over time in both groups

Both groups demonstrated a significant reduction in NRS scores and WOMAC subscale scores at 1 and 6 months compared with baseline (*p* < 0.05). Temporally, clinical scores reached their lowest values at one month. While a mild symptomatic rebound was observed by the sixth month, scores in both groups remained significantly lower than pre-procedure baseline values (Table 2) (Figs. 3 and 4).

**Table 2:** Comparison of NRS and WOMAC score changes between groups

	IASI (*n* = 50) Median (IQR)	IASI+IPACK (*n* = 48) Median (IQR)	*p*
NRS			
T_0_-T_1_	3,00 (2,25)	4,00 (2,00)	0,015
T_0_-T_2_	2,00 (2,00)	2,00 (2,00)	0,903
T_1_-T_2_	-1,00 (1,00)	-2,00 (2,00)	0,001
WOMAC(Pain)			
T_0_-T_1_	5,00 (3,00)	5,00 (2,00)	0,436
T_0_-T_2_	4,00 (2,50)	4,0 (2,00)	0,640
T_1_-T_2_	-1,00 (2,25)	-1,00 (2,0)	0,260
WOMAC(Stiffness)			
T_0_-T_1_	3,00 (1,00)	4,00 (1,00)	0,080
T_0_-T_2_	3,00 (2,00)	3,00 (1,75)	0,787
T_1_-T_2_	-1,00 (1,00)	-1,00 (1,75)	0,124
WOMAC(Function)			
T_0_-T_1_	7,50 (3,00)	11,00 (2,75)	0,000
T_0_-T_2_	3,00 (3,25)	6,00 (3,00)	0,000
T_1_-T_2_	-4,00 (4,0)	-6,00 (3,00)	0,002
WOMAC(Total)			
T_0_-T_1_	16,00 (4,25)	20,00 (5,00)	0,000
T_0_-T_2_	10,00 (5,25)	12,00 (4,00)	0,024
T_1_-T_2_	-6,00 (5,00)	-8,00 (3,00)	0,000

*IQR* Interquartile Range, *IASI* Intra Articular Steroid Injection, *IPACK* Infiltration between Popliteal Artery and Capsule of the Knee

T0 = Before the procedure, T1 = 1st month of the procedure, T2 = 6th month of the procedure

### Between-group comparisons

The IASI+IPACK cohort exhibited a significantly greater reduction in NRS scores at one month compared to the IASI group (*p* < 0.05). Although both groups showed improvement at 6 months, the overall change from baseline was similar between them. However, the increase in scores from month 1 to month 6 was more pronounced in the IASI+IPACK group (*p* < 0.05) (Table 2) (Figs. 3 and 4).

In the WOMAC assessment, no significant differences were found between treatment groups in the changes of Pain and Stiffness subscales. Significant differences were observed in WOMAC Function and WOMAC Total scores. Group IASI+IPACK showed significantly greater improvement than Group IASI in the transition from baseline to both 1 month and 6 months (*p* < 0.05). Correlation analysis did not show significant relationship between symptom duration and treatment efficacy (changes in NRS and WOMAC scores) at any post-procedure time point in either group (*p* > 0.05) (Table 2) (Figs. 3 and 4).

Paracetamol consumption decreased significantly in both groups at 1 month and 6 months post-procedure compared to baseline (*p* < 0.05). Although consumption at 6 months slightly increased compared to 1 month, it remained below baseline levels in both groups. When comparing the two groups, no statistically significant difference was found between the treatment groups regarding the changes in monthly paracetamol usage (*p* > 0.05). However, detailed patient-level data regarding the number of patients using rescue medication and the frequency of intake were not consistently available due to the retrospective design of the study.

## Discussion

This retrospective cohort study evaluated the efficacy and safety of adding an IPACK block to intra-articular steroid injection in patients with advanced knee osteoarthritis. We hypothesized that adding an IPACK block to IASI would provide greater improvement in pain and function compared to IASI alone. Our findings suggested that both treatment modalities provided significant improvements in pain and functional scores at 1 and 6 months post-procedure compared to baseline. Although scores increased slightly at 6 months compared with the marked reduction observed at 1 month, they remained significantly lower than baseline values.

An important finding of this study was the more pronounced increase (rebound) in scores from the 1st month to the 6th month in the Group IASI+IPACK compared to the control group. Our findings are consistent with previous studies demonstrating the additional analgesic benefit of targeting posterior knee structures. Similar rebound pain phenomena have been reported in studies evaluating peripheral nerve blocks, including IPACK and adductor canal blocks, particularly following the resolution of the block effect [15]. Although this might initially appear as a disadvantage, it can be attributed to the more pronounced early improvement provided by the IPACK block. In the IASI+IPACK group, pain and function scores dropped to much lower levels at 1 month compared to the group receiving only steroids. As the pharmacological effects of the steroid and block gradually diminished, symptoms tended to increase again over time.

It is well established that exercise plays a key role in the management of knee osteoarthritis. Furthermore, patients who experienced substantial early pain relief may have gradually increased their physical activity levels, potentially contributing to the relative rise in scores observed toward the sixth month. Adherence to the home exercise program may also have contributed to the observed clinical improvements, particularly in functional outcomes. However, as compliance was not systematically recorded due to the retrospective design, its exact impact on the results could not be determined. Despite this, the fact that WOMAC Function and Total scores in the IASI+IPACK group were still significantly better than those in the IASI group at the end of 6 months supports the long-term efficacy of this combined approach.

The IPACK block is a relatively novel technique, first described in 2012, which has since gained increasing popularity. It is frequently utilized as part of multimodal analgesia protocols in postoperative pain management. Although its efficacy in this context has been demonstrated in randomized controlled trials [11], most existing evidence originates from postoperative settings, and data regarding its use in chronic non-surgical knee osteoarthritis remain limited. Edwards et al. reported that an IPACK block with added steroids reduced chronic pain and improved physical function for approximately two months in patients awaiting knee arthroplasty [13]. Consistent with these findings, our study showed the best results at 1 month, followed by an increase in scores toward the 6th month. Previous studies have shown in the literature that the addition of steroids to local anesthetics in peripheral nerve blocks prolongs the duration of analgesia through anti-inflammatory effects, suppression of ectopic discharges, and inhibition of C-fiber transmission [14]. Indeed, findings from studies on IPACK blocks suggest that adding steroids may extend efficacy. For instance, one study demonstrated that the combination of IPACK and adductor canal blocks with steroids not only improved postoperative pain management after total knee arthroplasty but also contributed to the reduction of rebound pain and chronic postsurgical pain one year after surgery [15].

Most interventional pain treatments for chronic knee pain target the genicular nerves. While interventions targeting these nerves are often successful, their effects may be limited as they mainly innervate the anterior and lateral aspects of the knee. The effectiveness of the IPACK block may be attributed to its ability to target the posterior articular branches of the knee joint, which are not sufficiently addressed by these approaches. The IPACK block differs in that it blocks pain originating from the posterior compartment. A cadaveric study reported that the injectate spreads not only posteriorly but also anterolaterally and anteromedially [10]. Although one comparative study found genicular blocks to be more effective than IPACK blocks on postoperative pain [16], another study reported that adding an IPACK block to a genicular block provided improved analgesia and enhanced recovery compared to the genicular block alone [17].

From a safety perspective, the IPACK block was well tolerated in our cohort. While rare complications such as vascular injury or transient foot drop have been reported in the literature [18–20], the vast majority of studies report no major adverse events [21, 22]. Consistent with these findings, we observed no major complications associated with the block in our study.

Our study has several limitations. The primary limitation is its retrospective design, which lacks randomization and carries a risk of selection bias. Furthermore, certain variables such as patients’ physical activity levels, adherence to the home exercise program, detailed rescue medication use, and quality of recovery measures were not systematically recorded or analyzed. Additionally, although quality of recovery is an important outcome in contemporary studies, our study focused primarily on pain and functional outcomes as the main clinical endpoints in a non-surgical chronic pain population. The absence of recorded sensory tests to confirm block success in the early post-procedure period, as well as the assessment of pain using a total score rather than differentiating between anterior and posterior compartments, represent additional limitations.

Despite these limitations, a key strength of our study is that it is among the early investigations evaluating the role of the IPACK block in the non-surgical management of chronic knee pain. Future prospective, randomized, placebo-controlled studies are needed to better define the efficacy of the IPACK block in chronic knee osteoarthritis. Additionally, comparative studies with other interventional techniques, such as genicular nerve block or ablation, would further contribute to the literature.

From a clinical perspective, the addition of the IPACK block may provide enhanced short-term pain relief, although its long-term benefits should be interpreted with caution.

## Conclusion

In conclusion, adding an IPACK block to intra-articular steroid injection appears to be a safe and effective option for pain management in advanced knee osteoarthritis. The combined treatment provided greater benefits, particularly in physical function and early pain relief, compared to steroid injection alone. This approach may be considered a valuable alternative for patients who are awaiting total knee arthroplasty, are at high surgical risk, or are unwilling to undergo surgery. However, these findings should be interpreted with caution due to the retrospective design, lack of randomization, and the absence of certain variables such as exercise compliance, physical activity levels, and detailed medication use.

## Data Availability

The datasets used and/or analysed during the current study are available from the corresponding author on reasonable request.

## Abbreviations

BMI: Body Mass Index
IASI: Intra-articular Steroid Injection
IPACK: Infiltration between the Popliteal Artery and the Capsule of the Knee
MRI: Magnetic Resonance Imaging
NRS: Numeric Rating Scale
OA: Osteoarthritis
SPSS: Statistical Package for the Social Sciences
STROBE: Strengthening the Reporting of Observational Studies in Epidemiology
WOMAC: Western Ontario and McMaster Universities Osteoarthritis Index
